# Relationship between Biochemical Bone Markers and Bone Mineral Density in Patients with Phenylketonuria under Restricted Diet

**Published:** 2013-12-31

**Authors:** Hala M. Koura, Sherif M. Zaki, Nagwa A. Ismail, Emad E. Salama, Dalia H. El Lebedy, Laila K. Effat

**Affiliations:** 1 Department of Pediatrics, National Research Centre; 2 Department of Anatomy, Faculty of Medicine; 3 Clinical Pathology Department, National Research Centre; 4 Department of Molecular Genetics, National Research Centre, Cairo University, Cairo, Egypt

**Keywords:** Phenylketonuria, PKU, Bone Mineral Density, RANKL, Osteocalcin, Bone Mineral Content

## Abstract

***Objective:*** Most of phenylketonuria (PKU) develops bone turnover impairment and low bone mineral density (BMD). Measurements of BMD reﬂect only bone mineral status but not the dynamics of bone turnover. Bone markers are a noninvasive tool useful for the assessment of bone formation and bone resorption processes. Our study was to assess the levels of bone markers in PKU in order to select a screen marker and detect the most specific marker which can be combined with BMD for appropriate follow up.

***Methods:*** Thirty three classic PKU patients were studied. BMD and bone mineral content (BMC) were measured. Total alkaline phosphatase (ALP), osteocalcin (OC) and carboxy-terminal propeptide of type I collagen (CICP), osteoprotegerin (OPG), receptor activator of nuclear factor κβ ligand (RANKL) and Deoxypyridinoline (DPD) were measured.

***Findings*** ***:*** Nineteen (57.6%) male and fourteen (42.4 %) female PKU patients were involved in the current study. Their mean age was 8.4±4.6 yrs and the age range 3-19 yrs. The control group consisted of twenty two (52.4%) males and twenty (47.6%) females. Their mean age was 8.5±3.3 yrs and th age range 2-17 yrs. Using the Z score values, there was a significant decrease of total BMC (TBMC-Z), BMD of the femoral neck BMD-FN-Z, BMD of lumbar vertebrae (BMD-L-Z), BMD-FN and DPD while RANKL increased. There was a negative correlation between CICP and TBMC and between CICP and BMD-L in these patients. Also, a negative correlation between ALP and TBMC and between ALP and BMD-L was observed. It was concluded that the ALP provides a good impression of the new bone formation in the PKU patients and it has a highly significant negative correlation with the many parameters of the bone mineral status beside the wide availability of inexpensive and simple methods. So a screening test and/or follow up for the PKU patients using ALP would be available. Once the level of ALP decrease is detected, one can combine it with BMD to explore the bone mineral status and with specific bone markers (OC, RANKL and DBD), to verify the dynamics of bone turnover.

***Conclusion:*** This schedule will reduce the risk of exposure of these patients to the risk hazards of DXA and limit its use only to a limited number of the highly suspected cases.

## Introduction

Phenylketonuria (PKU) is a disorder in which elimination diets are the only known therapy, which reverses many clinical manifestations of the acute phase. Unfortunately, most of the patients develop bone turnover impairment and low bone mineral density (BMD)^[^^1^^]^. The reduced BMD may be inherent to PKU and/or secondary to its dietary treatment^[^^2^^]^. Measurements of BMD reﬂect only bone mineral status but not the dynamics of bone turnover^[^^1^^]^.

 Biochemical bone markers are a valuable noninvasive tool useful for the assessment of bone formation and bone resorption processes^[^^3^^]^. They have been developed over the past 20 years^[^^4^^]^. Markers of bone formation are either generated from newly synthesized collagen such as carboxy-terminal propeptide of type I collagen (CICP) or osteoblast-related proteins such as osteocalcin (OC) and alkaline phosphatase^[^^5^^]^. The majority of bone resorption markers are degradation products of bone collagen such as hydroxyproline. Other markers of bone resorption include non-collagenous matrix proteins such as bone Sialoprotein, or osteoclast-specific enzymes like tartrate-resistant acid phosphatase^[^^5^^]^. Other important resorption markers are N-terminal cross-linking telopeptide of collagen type I (NTX), C-terminal cross-linking telopeptide of collagen type I (CTX), and tartrate-resistant acid phosphatase isoform-5b (TRACP-5b)^[^^6^^]^.

 Increases in bone resorption marker levels have been documented in PKU patients^[^^7^^]^. Such markers can also be useful in selected cases to improve the assessment of individual fracture risk when BMD measurement by itself does not provide a clear answer. The combined use of BMD measurement and bone markers is likely to improve the assessment of the risk of fractures^[^^8^^]^.

 There are a large number of bone markers and the use of all these markers for assessment of PKU patients is tedious and economically unwise. So, the aim of the study was to assess the levels of some bone formation and resorption markers in PKU patients in order to select a screen marker and to detect the most specific marker which may combine with BMD for appropriate follow up in these patients to prevent osteopenia and osteoporosis in later life.

## Subjects and Methods

We enrolled 33 classic PKU patients from the Association of Genetic and Metabolic Disorders and studied in the pediatrics clinic, National Research Centre (NRC). 

 The selected patients were receiving a low protein diet consisting of low protein breads and pastas (wheat starch, metamucil and methylcellulose), fresh fruits and vegetables^[^^9^^]^. Carbohydrates and fat as well as vitamins, minerals and trace elements were calculated according to the national councils^[^^10^^]^.

 Informed consents were taken from the controls and the parents of the PKU children according to the World Medical Association (WMA) Declaration of Helsinki Sixth revision guidelines^[^^11^^]^ and according to the guidelines of the ethics committee of NRC, Egypt.

 Forty four age- and sex-matched controls were studied. All the control children were healthy with no known medical illness. Anthropometric measurements were taken following standardized techniques^[^^12^^]^. The weight was measured with a digital balance and the height with a stadiometer. 

 Norland densitometer (XR-46, USA) Rev. 3.9.6 /2.3.1 Was used for assessment of BMD and bone mineral content (BMC)^[^^13^^]^. An abnormal dual-energy x-ray absorptiometry (DXA) was defined as more than one SD below the normal mean, expressed as Z-score <-1. “Low bone mass for chronological age” is the preferred term when the BMC or BMD Z-score is less than or equal to -2.0^[^^14^^]^. 

 Plasma phenylalanine (Phe) level was measured by fluorometeric method which is a quantitative and precise method to select the controlled diet patients^[^^15^^]^. Also, liver function test was done to rule out liver disease.

 Blood and urinary markers of bone formation and resorption were measured to evaluate bone remodeling and turnover^[^^16^^]^. 10 ml of venous blood, fasting morning sample, was withdrawn on EDTA-vacutainer collection tubes from each patient. Serum was separated by centrifugation and stored at -20^o^C, not more than 2 hours, till assay. For deoxypyridinoline (DPD), random urine samples were collected and stored at -20^o^C till assay. 

 Serum total alkaline phosphatase (ALP), OC, and CICP were measured as bone formation markers. CICP was also measured as an indicator of bone collagen production and growth^[^^17^^]^. ALP was assayed using Olympus autoanalyzer AU400(Olympus Diagnostica, Japan).

**Table 1:** Comparison of the bone mineral status between the control and Phenylketonuria groups

**Variable**	**Group **	**n**	**Mean (SD)**	**t-test**	***P.*** ** value**
**TBMC (gm)**	Control	42	1269.3 (557.3)	1.4	0.1
	Phenylketonuria	33	1072.7 (596.7)		
**TBMC-Z **	Control	42	1.1 (1)	4.5	<0.001
	Phenylketonuria	33	-0.1 (1.2)		
**BMD-FN** ** (gm/m** ^2^ **)**	Control	42	0.7 (0.1)	3.7	<0.001
	Phenylketonuria	33	0.5 (0.1)		
**BMD-FN-Z **	Control	42	0.02 (0.4)	5	<0.001
	Phenylketonuria	33	-0.6 (0.7)		
**BMD-L** ** (gm/m** ^2^ **)**	Control	42	0.5 (0.1)	1.5	0.1
	Phenylketonuria	33	0.5 (0.1)		
**BMD-L-Z **	Control	42	0.06 (0.6)	1.6	0.01
	Phenylketonuria	33	-0.3 (0.6)		

SD: Standard Deviation; TBMC: Total Body Mineral Content; BMD-FN: Body Mineral Density of the Femoral Neck; BMD-L: Body Mineral Density of Lumbar Vertebrae

OC was measured by Enzyme-Linked Immunosorbent Assay (ELISA) kit, catalogue # KAP1381 from BioSource Europe SA, Nivelles, Belgium. CICP was measured in serum by MicroVue CICP ELISA kit, catalogue # 8003, Quidel Corporation, San Diego, CA, USA.

 Markers of osteoclastic activity, Osteopro-tegerin (OPG) and Receptor activator of nuclear factor κβ ligand (RANKL) were measured. Furthermore, OPG was measured because it is a major inhibitor of bone resorption^[^^18^^]^. It was measured in serum using Osteoprotegerin Human ELISA Kit, catalogue # RD194003200, BioVendor Laboratory Medicine, Inc., Czech Republic. RANKL was measured in serum by using ELISA kit catalogue # BI-20422H, Biomedica Medizin-produkte GmbH & Co KG, Wien, Austria.

 As a marker of bone collagen breakdown, DPD was measured in urine by MicroVue DPD ELISA kit, catalogue # 8007, Quidel Corporation, San Diego, CA, USA.

 The results were analyzed statistically using SPSS version 20. Power calculation for the sample size was done. The study was stated as having 80% power. Results were expressed as means±SD. The independent t-test was used to compare between the bone markers and between the different parameters of the bone mineral status of the PKU patients and those of the control group. The Pearson correlation coefficient was used to determine the relationship between the bone markers and the quantitative variables of the bone mineral status in the PKU patients. The quantitative data were examined by Kolmogrov Smirnov test for normality. Significance was considered at *P*-value <0.05.

## Findings

Nineteen (57.6%) male and fourteen (42.4%) female PKU patients were involved in the current study. Their mean age was 8.4±4.6 yrs, range 3-19 yrs. and median 7 yrs. The control group consisted of twenty two (52.4%) males and twenty (47.6%) females. Their mean age was 8.5±3.3 yrs, range 2-17 yrs and median 8 yrs. No difference was detected between the mean age in patients and control group. The mean level of phenylalanine in PKU patients was 12.3±0.95 mg/dl. The mean weight and height of the PKU patients was 28±13.5 kg and 121.6±23.3 cm respectively, while that of the controls was 33±15.6 kg and 128.4±18.7 cm respectively.

Table 1 shows the bone mineral status of the control and the PKU group.

 Using the Z score values, there was a statistically significant decrease of the total body mineral content (TBMC-Z) (*P*=0.000), bone mineral density of the femoral neck (BMD-FN-Z) (*P*=0.000) and bone mineral density of L_2_-L_4_ lumbar vertebrae (BMD-L-Z) (*P*=0. 01) of the PKU patients when compared to their fellows of the control group. The BMD-FN-Z (t=5) changes were more sensitive when compared to that of the TBMC-Z (t=4. 5) and that of the BMD-L-Z (t=1.6).

 Also there was a statistically significant decrease of the BMD-FN (*P*=0.000) and that of the PKU patients when compared to that of the control group. On the other hand, statistically insignificant changes in the TBMC (*P*=0.1) and BMD-L (*P*=0.1) of the PKU patients when compared to those of the control group (Table 2).

**Table 2:** Comparison of the bone markers between the control and the PKU groups

**Parameter**	**Group **	**n**	**Mean (SD)**	**t-test**	***P.*** ** value**
**Carboxy-terminal propeptide of type I collagen (** **ng/ml** **)**	Control	42	270.7 (89.6)	0.5	0.5
	Phenylketonuria	33	283.4 (114.7)		
**Alkaline phosphatase (U/L)**	Control	42	152.0 (43.1)	2.9	0.005
	Phenylketonuria	33	121.6 (46.0)		
**Osteocalcin ** **(mg/dl)**	Control	42	43.4 (34.5)	6.1	<0.001
	Phenylketonuria	33	13.9 (12.9)		
**Receptor activator of nuclear factor κβ ligand (RANKL) (** **ng/ml** **)**	Control	42	0.1 (0.07)	19.7	<0.001
	Phenylketonuria	33	1.0 (0.2)		
**Osteoprotegerin (OPG) ** **(ng/ml)**	Control	42	3.3 (2.3)	1.7	0.09
	Phenylketonuria	33	4.0 (0.8)		
**Deoxypyridinoline (** **mmol/mmol creatinine** **)**	Control	42	68.1 (30.7)	4.3	<0.001
	Phenylketonuria	33	32.3 (15.0)		
**OPG /RANKL ratio**	Control	42	0.56 (0.06)	12	0.09
	Phenylketonuria	33	0.27 (0.13)		

SD: Standard Deviation

 Regarding the bone formation markers, there was a significant decrease of mean OC (*P*=0.000) and ALP (*P*=0.005) of the PKU patients in comparison to those of the control group. The OC changes were more significant (t=6.1) when compared to those of ALP (t=2.9). On the other hand, the mean CICP of the PKU patients was statistically insignificant (*P*=0.5) when compared to that of the control group. 

 Regarding the osteoclastic activity markers, there was a significant increase of mean RANKL (*P*=0.000) of the PKU patients in comparison to that of the control group. On the other hand, the mean OPG of the PKU patients was statistically insignificant (*P*=0.09) when compared to that of the control group. 

 Regarding the bone collagen breakdown marker (DPD), there was a statistically significant decrease in the mean in the PKU patients (*P*=0.000) in comparison to that of the control group. 

Table 3 hows correlations between the bone markers and the parameters of the bone mineral status in the PKU patients. 

 There was a statistically high significant negative correlation between the CICP and the TBMC from one side and between the CICP and the BMD-L from the other side. Similarly, there was a highly significant negative correlation between the ALP and the TBMC from one side and between the ALP and the BMD-L from the other side. Finally, there was a statistically significant correlation between the DBD and the BMD-L. No significant correlation was observed between DBD and abundant form of collagen found in bone^[^^17^^]^. When using CICP as a marker for bone formation, statistically insignificant changes were observed in the PKU patients when compared to those of the control group.

**Table 3:** Correlations between the bone markers and parameters of the bone mineral status in the PKU group

**Bone Markers**	**Test**	**TBMC**	**TBMC-Z**	**BMD-FN** ** (gm/m** ^2^ **)**	**BMD-FN-Z**	**BMD-L** ** (gm/m** ^2^ **)**	**BMD-L-Z**
**CICP**	**Pearson Correlation**	-0.51^**^	0.003	-0.29	-0.029	-0.49^**^	-0.16
	**Sig. (2-tailed)**	0.002	0.9	0.1	0.8	0.004	0.3
**Alkaline phosphatase**	**Pearson Correlation**	-0.54^**^	-0.16	-0.28	-0.096	-0.59^**^	-0.19
	**Sig. (2-tailed)**	0.001	0.3	0.1	0.6	0.000	0.2
**Osteocalcin**	**Pearson Correlation**	-0.0	-0.12	-0.04	-0.091	-0.009	-0.17
	**Sig. (2-tailed)**	0.9	0.3	0.8	0.6	0.9	0.3
**RANKL**	**Pearson Correlation**	-0.03	0.05	-0.15	0.046	-0.18	-0.03
	**Sig. (2-tailed)**	0.8	0.7	0.3	0.8	0.3	0.8
**Osteoprotegerin**	**Pearson Correlation**	0.07	0.17	0.09	0.059	0.07	-0.01
	**Sig. (2-tailed)**	0.6	0.3	0.5	0.7	0.6	0.9
**Deoxypyridinoline**	**Pearson Correlation**	-0.31	-0.3	-0.27	-0.124	-0.36^*^	-0.07
	**Sig. (2-tailed)**	0.07	0.08	0.1	0.5	0.03	0.6

CICP: carboxy-terminal propeptide of type I collagen; RANKL: Receptor activator of nuclear factor κβ ligand; TBMC: Total body mineral content; BMD-FN: Body mineral density of the femoral neck; BMD-L: Body mineral density of lumbar vertebrae

In spite of this finding, it was observed from our correlation studies that the CICP had a statistically high significant negative correlation with TBMC and BMD-L. Although this correlation is very beneficial, we cannot rely on it alone as the clinical relevance of CICP in the evaluation of metabolic bone diseases is viewed with skepticism^[^^26^^]^. It does not appear to be a sensitive index of bone formation^[^^27^^]^. 

 Regarding the osteoclastic activity markers, the RANKL of the PKU patients increased significantly in comparison to that of the control group. The OPG/receptor activator of nuclear factor κB (RANK)/RANKL system is a key regulatory system in bone metabolism^[^^28^^]^. The increased RANKL level of the PKU patients in the current study may predispose them to the risk of osteopenia as the complete absence of RANKL leads to osteoporosis^[^^29^^]^. 

 The other osteoclastic activity marker is OPG which was statistically insignificant in PKU patients when compared to that of the control group. Its absence, something not present in our study, leads to osteoporosis due to unbalanced activity of RANKL and enhanced osteoclast formation^[^^20^^]^.

 The OPG/RANKL ratio was lower in the PKU patients compared to that of the control group (*P*=0.09). The decrease of this ratio may account for an enhanced ability of preosteoblastic cells to support osteoclast development, leading to the imbalance between bone formation and resorption and rapid bone loss^[^^30^^]^.

 The DPD decreased significantly in the PKU patients in comparison to that of the control group. It is formed during the extracellular maturation of fibrillar collagens, bridges several collagen peptides and stabilizes the collagen molecule^[^^5^^]^. During bone resorption, it is broken down and released into the circulation and urine^[^^5^^]^. DBD is currently viewed the best index for assessing bone resorption^[^^31^^]^. In the current study DBD has a significant negative correlation to the BMD-L.

 Patients with developmental or psychomotor disturbance were excluded. None of our patients had a history of immobilization or movement limiting disease. Also we ruled out any patients with known liver disease.

## Conclusion

 It could be concluded that the ALP, once liver disease is ruled out, provides a good impression of new bone formation in PKU patients and it has a highly significant negative correlation with the many parameters of the bone mineral status, beside the wide availability of inexpensive and simple methods. So we can do a screening test and/or follow up for PKU patients using the ALP. Once the decrease of ALP level is detected, one can combine it with BMD to explore the bone mineral status and the specific bone markers (OC, RANKL and DBD) to verify the dynamics of bone turnover. This schedule will reduce the risk of exposure of these patients to the risk hazards of DXA and limit its use only to a limited number of the highly suspected cases. In addition, this schedule will evaluate both the bone mineral status and the dynamics of bone turnover. 

 We recommend more widespread studies to include other bone formation and resorption markers, which were not included in our studies for economic reasons, to draw a final conclusion for the most preferable use of bone markers in cases of ALP elevation in PKU patients.
